# 3D-Printed Detector Band for Magnetic Off-Plane Flux Measurements in Laminated Machine Cores

**DOI:** 10.3390/s17122953

**Published:** 2017-12-19

**Authors:** Georgi Shilyashki, Helmut Pfützner, Martin Palkovits, Andreas Windischhofer, Markus Giefing

**Affiliations:** Institute of Electrodynamics, Microwave and Circuit Engineering, TU Wien, Gußhausstraße 25/354, 1040 Vienna, Austria; helmut.pfuetzner@tuwien.ac.at (H.P.); martin.palkovits@tuwien.ac.at (M.P.); andreas.windischhofer@tuwien.ac.at (A.W.); e1425841@student.tuwien.ac.at (M.G.)

**Keywords:** flux distribution, off-plane flux, machine cores, magnetic sensors

## Abstract

Laminated soft magnetic cores of transformers, rotating machines etc. may exhibit complex 3D flux distributions with pronounced normal fluxes (off-plane fluxes), perpendicular to the plane of magnetization. As recent research activities have shown, detections of off-plane fluxes tend to be essential for the optimization of core performances aiming at a reduction of core losses and of audible noise. Conventional sensors for off-plane flux measurements tend to be either of high thickness, influencing the measured fluxes significantly, or require laborious preparations. In the current work, thin novel detector bands for effective and simple off-plane flux detections in laminated machine cores were manufactured. They are printed in an automatic way by an in-house developed 3D/2D assembler. The latter enables a unique combination of conductive and non-conductive materials. The detector bands were effectively tested in the interior of a two-package, three-phase model transformer core. They proved to be mechanically resilient, even for strong clamping of the core.

## 1. Introduction

In the past, it was well accepted that flux distribution in cores of transformers or rotating machines can be considered as a two-dimensional magnetization problem. However, recent studies have shown that laminated soft magnetic cores may exhibit complex 3D flux distributions with pronounced normal fluxes perpendicular to the magnetization plane. For instance, transformer cores show a specific design of several packages of different width, apart from local inhomogeneities of magnetic flux in the rolling direction [1,2]. This may yield strong off-plane fluxes, especially within and close to overlap regions of corners and T-joints [3]. In the case of shunt reactors, the specific combination of circular limbs and rectangular yokes is a source of certain off-plane fluxes. For induction machines (generators and motors), axial, off-plane flux exists, due to stray fluxes [4] as well as due to eccentricity faults [5]. 

As a general tendency, off-plane fluxes may lead to increases of local core losses, as well as of local magnetostriction values [3] and magneto-static forces [6]. This means that the detection of local off-plane inductions *B*_ND_ in normal direction tends to be essential for improving core designs in order to reduce losses and audible noise.

Usually, numerical modelling is used for calculations of entire local 3D flux distributions of cores, including the off-plane fluxes. Calculations are preferred by researchers and industrialists among others due to the existence of novel, powerful and easy to use commercial software tools such as COMSOL Multiphysics, FLUX 2D/3D etc. The latter are based on Finite Elements Methods (FEM). Some other approaches use Magnetic Equivalent Circuits (MEC) or the novel Magnetic Anisotropic Circuit Calculation (MACC) [7]. Results for transformer cores are reported in [8,9], cores of rotating machines in [4,10,11], and shunt reactors in [12]. However, apart from high computational times, a significant drawback of all numerical methods is the lack of accurate material data, e.g., non-linear permeability functions in the transverse and in the normal direction. The latter may be a source of significant deviations of calculated local induction values from real ones.

As an alternative to calculations, fluxes in the off-plane direction can be measured by means of different experimental methods. Generally, off-plane flux measurements tend to be laborious and are performed mostly on specially prepared model cores of laminated machines. A well-known method for flux detection is the usage of Hall-effect sensors, located at different core locations, as performed in [13] for a synchronous generator. However, even the thinnest sensors are about 1 mm thick and appropriate for measurements only on the surface, not in the core interior. Another possibility would be the usage of single turn normal search coils of thin wire stuck directly onto laminations, as often used for transformer cores [14,15]. However, these measurements are linked with high expenditure of manual work, requiring the opening of the core, preparation of laminations and re-stacking. Further, the thickness of wire, close to 100 µm, tends to influence the measured values *B*_ND_ in significant ways [16]. *B*_ND_ decreases in a non-linear way with an increase of sensor thickness, due to caused air gaps between laminations. Furthermore, the shifting of wires of the search coils and changing of their effective areas may appear during clamping. The most accurate measurements may be achieved by means of sputtered technique, as presented in [17] with a sensor thickness of about 1 µm. However, such measurements are extremely laborious and are restricted to small lamination sizes, as well as being very costly since they require special equipment. 

The subject of the present paper are novel detector bands for effective, fast and simple local off-plane induction measurements that overcome most of the above-mentioned drawbacks of existing sensors. Based on a concept as presented in [18], several off-plane sensors are printed on a very thin paper substrate of 20 µm thickness. The sensors are given by frame coils of several turns, thus representing 3D systems. In a previous work [16], they were manufactured semi-automatically by a mere 2D printing procedure, complemented by manually prepared contact bridges through holes. As a significant improvement, the current paper describes fully-automatic manufacturing by means of a novel 2D/3D assembler that combines conductive and plastic printing materials. While the previous work [16] described applications in the corner regions of a transformer core, the current paper presents an example of results for T-joint regions.

## 2. Manufacturing of Detector Bands

As already mentioned, with the manufactured detector bands, effective, fast and simple measurements should be possible. However, the requirements put on the bands are very high. They should yield high mechanical stability, being inserted into the interiors of laminated cores. As is well known, the latter are exposed in most cases to extremely strong clamping. Furthermore, the manufactured band should be of very low thickness, so as not to influence the magnetic flux and should, at the same time, be re-usable. In cases where data from several crucial regions of a laminated core are needed (e.g., as being practical for model cores), the band should be replaceable without being damaged. These demands are met by a novel concept [18] by means of a printing procedure, using a thin paper as a carrier and positioner. 

As a first attempt, we prepared an off-plane detector band, using mere 2D printing procedure of conductive ink on a thin kapton foil. In this way, as demonstrated in [16], a low total thickness of about 50 µm of the detector band can be achieved. However, for a sufficient signal strength of the detected relative low values of off-plane inductions—in particular, for low nominal magnetizations of the laminated cores—several turns for each printed off-plane sensor are needed. This cannot be attained with 2D printing. Therefore, in [16], through-holes for conductive return feed-lines were manually established. As a disadvantage, the holes cause artefacts, but, more importantly, they prevent an automatic manufacturing of detector bands.

**Figure 1:**
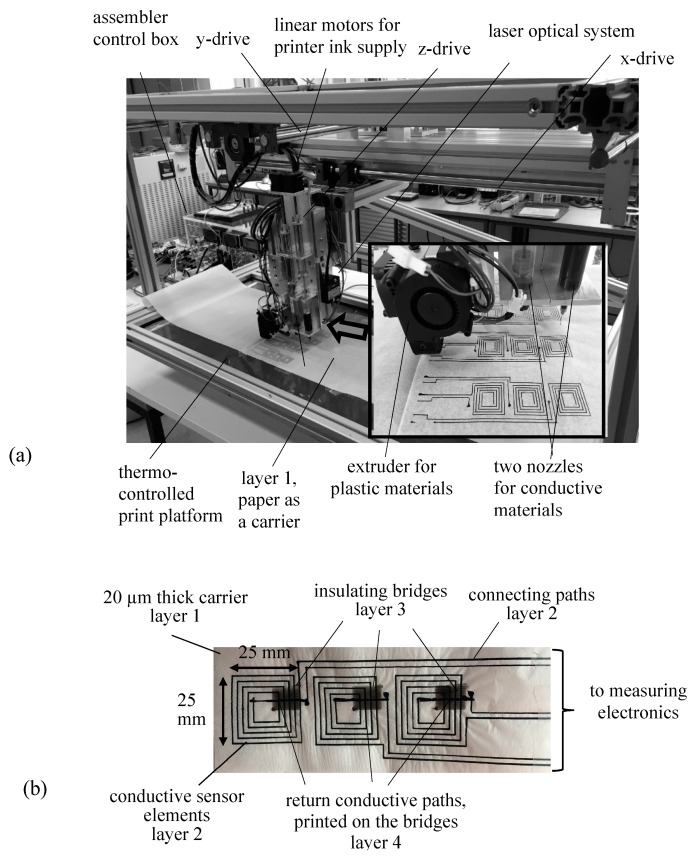
Manufacturing of detector bands: (**a**) 2D/3D printing assembler (insert: print sensors); (**b**) A manufactured band with three frame coil sensors for simultaneous detection of off-plane induction at three locations.

From its principles, a detector band with a multi-turn coil (Figure 1b) represents a 3D system. To attain automatic manufacturing, we developed a novel combined 3D/2D-assembler (Figure 1a) for simple, rapid, precise and automatic sensor preparation. The assembler enables a unique combination of conductive and non-conductive printing materials for manufacturing not only of off-plane sensors but of a variety of sensors, as used for a better understanding of magnetic performances of core interiors. 

The assembler consists of a large aluminum frame (length 1300 mm, width 900 mm and height 600 mm) and a scanning head, three-dimensionally driven by stepping motors (StepSyn, Type 103H7123-5740, Sanyo Denki), controlled by in-house developed software. On the scanning head, an extruder (BQ HeatCore DDG) is mounted, equipped with a 200 µm print nozzle needle. The latter is used for extruding of 3D plastic material (polylactic acid) for insulations and fixing of the to-be manufactured sensors. Next to the extruder, two additional in-house developed nozzles are mounted and controlled by extended software for disposal of conductive inks. The nozzles consist of a cartridge and a dispensing needle from the company Nordson EFD. Extrusion is activated by linear stepper motors (E35H4N-12-A03, Haydon Kek-Ametek). 

The application of the individual nozzles for conductive material into the assembling process is performed magnetically by software-controlled solenoids, mounted on the back side of the scanning head. To improve the printing quality, a large thermo-controlled glass platform (length 1000 mm, width 400 mm) is used. It enables the manufacture of long bands, as needed for large machine cores in order to keep contact regions outside. Finally, for local measurements of the thickness of the manufactured bands, a laser optical system (micro-epsilon, LD1605-20, Micro-Epsilon Messtechnik GmbH & Co. KG, Ortenburg, Deutschland) is mounted sideways on the scanning head. 

By means of the described 3D/2D-assembler, detector bands of different sizes, up to 1000 mm length and up to 400 mm width, can be manufactured in an automatic way. As intended in the concept [18], such detector bands can be left into the laminated cores of even very large machines for their entire lifetime, enabling monitoring of operation and effective failure detection. 

## 3. Design of Detector Band for Measurements of Off-Plane Induction

Figure 1b shows a manufactured detector band for off-plane induction detection with a triplet of frame coil sensors, enabling simultaneous measurements at three positions in laminated machine cores. The detector band represents a 3D system that consists of four layers. 

As a carrier (Layer 1), we used a paper band of 20 µm thickness. On the paper, as Layer 2, conductive paths of some µm thickness were printed, using one of the two nozzles for conductive material (compare Figure 1a). For the current application, as a printing material, we used a mixture of an electric paint, “bare conductive”, and water. As mentioned, for better signal strength, each sensor consists of five turns. This means that insulating bridges (Layer 3) are necessary. The latter were printed using the extruder for plastic material. A significant challenge was to print a heated plastic material over the ink, without damaging the electrical conductivity of the paths. Therefore, as a printing material, we used PLA (poly lactic acid) material, since the latter melts at much lower temperature than the conventional ABS (acrylonitrile butadiene styrene) plastic. The printed insulated bridge (Layer 3) represents the region of highest thickness. However, it can be assumed that this is balanced in clamped state due to the high elasticity and softness of the used PLA material. Finally, over the plastic material, a conductive Layer 4 using the same conductive ink was printed. 

Handles at both band ends (not shown in Figure 1b) enable precise positioning, inserting or shifting of the detector into the interior of a laminated machine core. Sensor contacts of increased thickness remain outside the core (Figure 1b), being mounted at one band end, thus not causing artifacts due to gaps. The induced voltages at the contacts are registered using a DAQ-device NI-USB6216. Both numerical integration of detected voltages and illustration of the off-plane induction values *B*_ND_ are performed in Matlab. 

The calculated effective cross section of each frame coil sensor was about 12 cm^2^. This relatively large cross section enables averaging over several grains of highly grain oriented materials. The electrical resistance of each frame coil sensor was about 20 kΩ, which proved to be insignificant for voltage detection with bigger than 1 MΩ input resistance. Using silver or nickel ink would reduce the resistance of the sensor significantly; however, this would increase detector costs. 

## 4. Example of a Result

The manufactured bands for off-plane detection are applicable in all types of laminated machine cores. In the current work, they were tested in the interior of a two-package, three-phase model transformer core, stacked from highly grain oriented material. 

As is well known, in transformer cores, high off-plane inductions *B*_ND_ appear direct in overlaps of corners and T-joints, but is restricted to small volumes [17]. In a previous work [16], we showed that additional normal flux arises in the vicinity of corner overlaps of high magnetic reluctance due to interlaminar air gaps, *B*_ND_ being up to the order of 0.1 T. This intensity is rather weak; however, it appears in rather large regions, thus showing practical relevance. In the here reported work, we studied the corresponding conditions in the vicinity of T-joint regions. 

**Figure 2:**
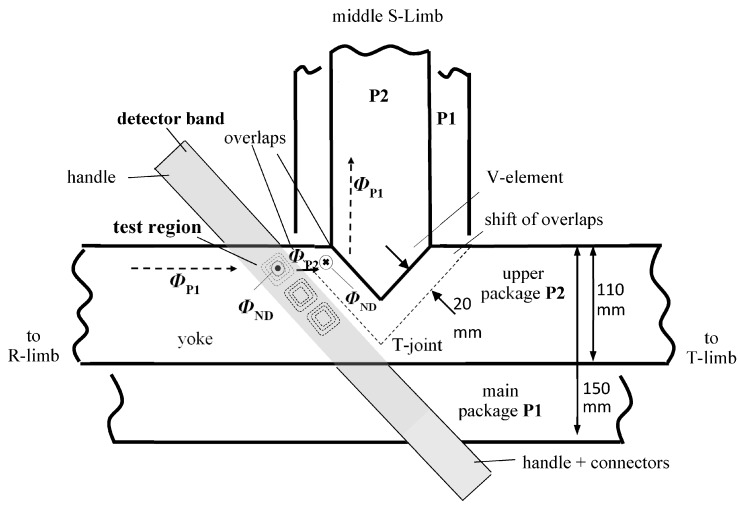
A rough outline of the T-joint region of a two-package model transformer core. The detector band is located between the wider main package P1 and the narrower upper package P2, very close to the overlap of P1. The paper presents a result only from the first uppermost frame-coil sensor, denoted as a “test region”.

**Figure 3:**
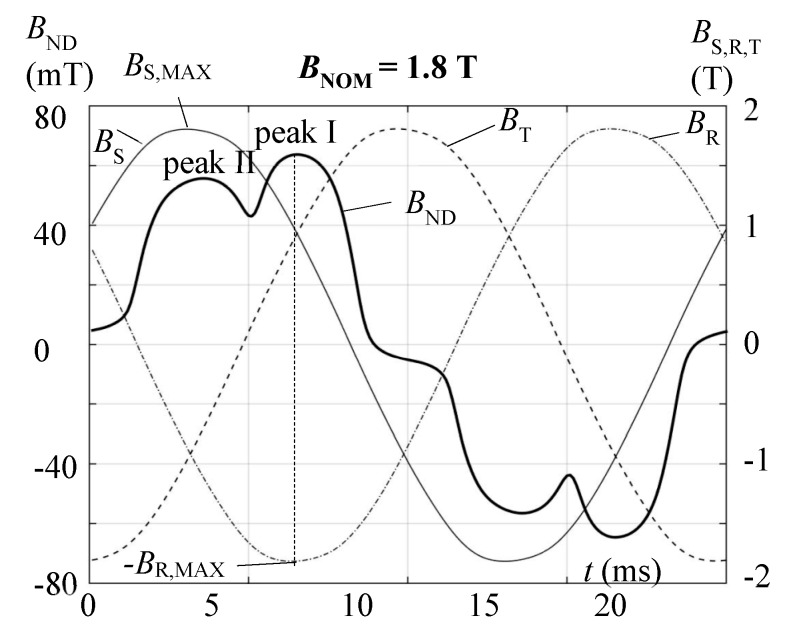
Detected off-plane induction *B*_ND_(*t*) in the “test region” (compare Figure 2) for *B*_NOM_ = 1.8 T. The courses of time of the inductions *B*_S_(*t*), *B*_T_(*t*), *B*_R_(*t*) of the three limbs, measured by means of search coils are illustrated as well.

Transformer cores are stacked typically of packages of different width. As a result, the T-joint exhibits a shift of overlaps [19], as sketched in Figure 2. As an example, the detector band was precisely inserted into the core interior, close to overlaps. Figure 3 shows result for a location that is entitled as “test region” in Figure 2, between two adjacent packages, i.e., main package P1 and outer package P2. 

Figure 3 shows the corresponding measured off-plane induction *B*_ND_(*t*) as a function of time. For a nominal core induction of 1.8 T, the depicted result exhibits a peak value of about 70 mT which is comparable with results from corners [16]. However, as a highly relevant feature, the waveform of *B*_ND_(*t*) tends to be strongly distorted which has impact on audible noise, apart from increasing local eddy currents losses.

Quite interestingly, *B*_ND_(*t*) shows two pronounced peaks I and II. The peak I of higher intensity appears for the time instant of maximal induction *B*_R,MAX_ in the outer R-limb. Peak II appears for the instant of maximal induction *B*_S,MAX_ in the middle S-limb, with a phase shift of 60° to the first peak. 

Figure 2 provides a simple schematic interpretation of peak II. Shortly before entering the T-joint, in the test region, a portion of main package flux *Ф*_P1_, coming from the (not depicted) left outer R-limb enters the upper package P2 as an off-plane flux *Ф*_ND_ that causes peak II. This flux “tries to avoid” the high reluctance of the overlap of P1. In a similar way, we expect that a part of the flux from the upper package *Ф*_P2_ re-enters the main package P1 in the region of shifted overlaps, avoiding the high reluctance of the overlap of P2. 

Peak I can be interpreted in an analogous way. As a whole, the results indicate the important role of the overlaps for the entire flux distribution of multi-package transformer cores.

## 5. Discussion and Conclusions

It should be stressed that the above results were detected with high reproducibility. The tests proved a high mechanical stability of the detector bands, even for extremely strong clamping of the core. Furthermore, re-arrangements of the sensors in different core locations proved to be easily possible without much expenditure of manual work. This means that the novel bands would allow studies of the entire off-plane flux distribution in a laminated core with much lower expenditure of manual work compared to conventional techniques. 

As a final conclusion, novel detector bands for off-plane flux measurements in the interior of laminated machine cores were developed. The latter consist of a thin paper (20 µm), used as a carrier, and an imprint of a triplet of multi-turn frame coils that represents a 3D system. The detectors are printed in an automatic way by an in-house developed 2D/3D assembler using a combination of conductive and non-conductive materials. The effective overall thickness of sensors can be assumed to depend strongly on the state of core clamping, and also on the elasticity of both the paper substrate and the printed layers as a function of temperature, which will be studied in future work. At any rate, even the mere 20 µm thick substrate creates an air gap that will decrease the local intensity of *B*_ND_. 

According to the tests, the detector bands enable effective, simple and fast measurements, overcoming the drawbacks of conventional methods with respect to laborious preparations. The latter promises a good scientific output of future applications, considering the high impact of off-plane flux on the magnetic performances of cores.
